# Predictors of futile recanalization after endovascular therapy in anterior circulation stroke with large core infarction

**DOI:** 10.3389/fneur.2025.1630438

**Published:** 2025-08-20

**Authors:** Qinhong Li, Chawen Ding, Boyu Chen, Zhenxuan Tian, Yujie Chen, Linyu Li, Nizhen Yu, Jiaxing Song, Jie Yang, Changwei Guo, Jiacheng Huang, Wenjie Zi, Zhao Yang

**Affiliations:** ^1^Department of Neurology, Yongchuan Hospital of Chongqing Medical University, Chongqing, China; ^2^Department of Neurology, The Second Affiliated Hospital of Chongqing Medical University, Chongqing, China; ^3^Department of Neurology, ChongGang General Hospital, Chongqing, China; ^4^Department of Neurology, Qujing First People's Hospital, Qujing, China; ^5^Department of Neurology, Sichuan Mianyang 404 Hospital, Mianyang, China; ^6^Department of Neurosurgery, Southwest Hospital, Third Military Medical University (Army Medical University), Chongqing, China; ^7^Department of Neurology, Xinqiao Hospital and The Second Affiliated Hospital, Army Medical University (Third Military Medical University), Chongqing, China

**Keywords:** anterior circulation large vessel occlusion, large core ischemic stroke, endovascular treatment, futile recanalization, modified Rankin scale

## Abstract

**Background:**

There is a lack of data to predict futile recanalization (FR) after endovascular treatment (EVT) in acute anterior circulation large vessel occlusion (ACLVO) with large core infarction.

**Methods:**

This analysis included patients from a national multicenter stroke registry (November 2021 to February 2023). Patients who achieved successful recanalization (expanded Thrombolysis in Cerebral Infarction [eTICI] score ≥2b) after EVT were categorized into two groups: meaningful recanalization (MR; 90-day modified Rankin scale [mRS] 0–3) and FR (mRS 4–6). Multivariate logistic regression was performed to identify independent predictors of FR.

**Results:**

Among 313 patients with successful recanalization, 171 (54.6%) experienced FR, and 142 (45.4%) achieved MR. Multivariate analysis showed that a higher baseline NIH Stroke Scale score (*p* < 0.001), older age (*p* < 0.001), elevated blood glucose (*p* = 0.003), poor collateral circulation (*p* = 0.004), and incomplete recanalization (eTICI 2b vs. 3; *p* < 0.001) were predictors of FR.

**Conclusion:**

In patients with ACLVO and large core infarction, age, hyperglycemia, baseline NIHSS, poor collaterals, and incomplete recanalization were independent predictors of FR. These findings may be used to guide treatment decisions and optimize management processes.

## 1 Introduction

Acute anterior circulation large vessel occlusion (ACLVO) with large core infarct accounts for approximately 20% of large vessel occlusion (LVO) strokes. It is a devastating cerebrovascular disease associated with high mortality and disability rates (1). Endovascular thrombectomy (EVT) with successful recanalization (expanded Thrombolysis in Cerebral Infarction [eTICI] ≥ 2b) is the proven effective treatment for ACLVO with large core infarction. This conclusion is supported by two landmark randomized controlled trials: SELECT2 (2023) (2) and ANGEL-ASPECT (2023) (3). Notably, both trials demonstrated that 53% (ANGEL-ASPECT) and 61.8% (SELECT2) of patients still experienced poor functional outcomes (90-day modified Rankin scale [mRS] 4–6) despite successful recanalization—a phenomenon termed futile recanalization (FR), which similarly affects other high-risk patient subgroups (4–6).

Patient characteristics including age, blood glucose, blood pressure, prehospital time, NIH Stroke Scale (NIHSS) scores, and other clinical variables have been established as independent predictors of FR across diverse populations. Predictive models integrating these factors can demonstrate accuracy and play a pivotal role in bridging fundamental research, imaging evaluation, and clinical decision-making (7–9). Despite the study of FR predictors being a global research priority, few studies have specifically focused on ACLVO with large core infarction selected solely by non-contrast CT (NCCT)-based Alberta Stroke Program Early CT Score (ASPECTS) scores of 3–5.

In this study, we analyzed data from the Prospective Multicenter Registry on Early Management of Acute Ischemic Stroke (MAGIC) to identify FR predictors in this specific population with successful recanalization after EVT.

## 2 Methods

### 2.1 Patient selection

Patients included in this study received treatment between November 1, 2021, and February 8, 2023. Our study was a subanalysis utilizing data from a nationwide, prospective registry in China, which enrolled patients presenting with acute large vessel occlusion and received standard treatment within 24 h of their last known well state in China (URL: http://www.chictr.org.cn. Uniform identifier: ChiCTR2100051664). Ethical approval for this study was granted by the institutional review board at the Second Affiliated Hospital of Army Medical University, and additional authorization was obtained from all participating centers’ ethics committees. From the originally published cohort (10), we conducted a subanalysis focusing exclusively on patients who met two additional criteria: (1) large ischemic core on NCCT (defined as an ASPECTS of 3–5); (2) eTICI ≥2b after EVT plus standard medical treatment (SMT).

### 2.2 Clinical evaluation and outcome

Baseline characteristics included: (1) demographic data; (2) stroke risk factors; (3) laboratory results; (4) stroke severity assessed by NIHSS (11); (5) collateral status evaluated using the American Society of Interventional and Therapeutic Neuroradiology/Society of Interventional Radiology collateral grading system (ASITN/SIR) (12); (6) stroke etiology classified according to the Trial of ORG10172 in Acute Stroke Treatment (TOAST) classification (13); (7) workflow durations; (8) EVT methodology; (9) recanalization grade (eTICI); (10) location of occlusion; (11) baseline core infarct volume determined by the NCCT-based ASPECTS; (12) Procedure-related complications.

An independent imaging core lab was blinded to treatment allocation and clinical results. Two trained neuroradiologists analyzed all imaging data; they independently evaluated the baseline NCCT-based ASPECTS and assessed occlusion sites using supplemental angiographic imaging. In cases of discrepancies, a third senior neuroradiologist adjudicated any discrepancies. Successful recanalization was defined as eTICI ≥2b (blood flow to greater than 50%). In our study, 90-day functional outcomes were assessed using the mRS through follow-up visits or telephone interviews conducted by trained and experienced local physicians. According to the 90-day mRS, patients were categorized into two groups: FR group (90-day mRS 4–6) and meaningful recanalization (MR) group (90-day mRS 0–3). We present details of the data elements in Table 1.

**Table 1:** Baseline characteristics in patients with meaningful and futile recanalization.

Characteristic	Overall (*n* = 313)	Meaningful recanalization	Futile recanalization	*P*
Age, median (IQR), y	68 (58–77)	64 (54–73)	73 (63–79)	<0.001
Sex, no. (%)				0.004
Men	191 (61.0)	99 (69.7)	92 (53.8)	
Women	122 (39)	43 (30.3)	79 (46.2)	
Baseline NIHSS score, median (IQR)	17 (13–20)	15 (12–18)	18 (14–21)	<0.001
Medical history, no. (%)				
Hypertension	191 (61)	81 (57.0)	110 (64.3)	0.188
Hyperlipidemia	70 (22.4)	33 (23.2)	37 (21.6)	0.735
Diabetes	48 (15.3)	18 (12.7)	30 (17.5)	0.234
Smoking	106 (33.9)	55 (38.7)	51 (29.8)	0.097
Atrial fibrillation	141 (45.0)	54 (38.0)	87 (50.9)	0.023
Blood pressure on admission,^a^ median (IQR), mmHg				
Systolic	145 (127–161)	143 (125–159)	146 (130–166)	0.078
Diastolic	86 (75–94)	88 (76–95)	85 (75–92)	0.365
Glucose, median (IQR),^b^ mmol/L	7.1 (5.8–9.1)	6.7 (5.6–7.8)	7.6 (6.2–10.1)	<0.001
Stroke causative mechanism, no. (%)				0.553
Large artery atherosclerosis	95 (30.4)	48 (33.8)	47 (27.5)	
Cardioembolism	178 (56.9)	76 (53.5)	102 (59.6)	
Other	11 (3.5)	6 (4.2)	5 (2.9)	
Unknown	29 (9.3)	12 (8.5)	17 (9.9)	
ASITN/SIR grade, no. (%)				<0.001
0–1	134 (42.8)	41 (28.9)	93 (54.4)	
2	121 (38.7)	66 (46.5)	55 (32.2)	
3–4	58 (18.5)	35 (24.6)	23 (13.5)	
Last seen well to imaging time, median (IQR), min	286 (159.0–454.5)	302 (178.8–513)	277 (147–437)	0.235
Last seen well to puncture time, median (IQR), min^c^	360 (240–553.5)	380 (258.5–599.3)	357 (233.5–542)	0.308
Last seen well to recanalization time, median (IQR), min^d^	440 (322–660)	440 (340-693)	440 (315–660)	0.634
Puncture to recanalization time, median (IQR), min^e^	88.3 (50–112.3)	81.0 (46.8–105.0)	94.3 (55.0–120.0)	0.066
First choice of endovascular treatment				0.595
Stent retriever thrombectomy	59 (18.8)	28 (19.7)	31 (18.1)	
Aspiration	182 (58.1)	83 (58.5)	99 (57.9)	
Balloon angioplasty and/or stenting	19 (6.0)	9 (6.3)	10 (5.9)	
Intra-arterial medication and/or mechanical fragmentation	4 (1.2)	0 (0)	4 (1.2)	
Swim	46 (14.7)	21 (14.8)	25 (14.6)	
Spontaneous reperfusion	1 (0.3)	0 (0)	1 (0.3)	
Pass stent no. (%)				0.171
<3	280 (90.3)	130 (92.9)	150 (88.2)	
≥3	30 (9.7)	10 (7.1)	20 (11.8)	
Pass aspiration, no. (%)				0.919
<3	273 (88.1)	123 (87.9)	150 (88.2)	
≥3	37 (11.9)	17 (12.1)	20 (11.8)	
Occlusion-site, no. (%)				0.055
Internal carotid artery	107 (34.2)	39 (27.5)	68 (39.8)	
M1 segment	174 (55.6)	89 (62.7)	85 (49.7)	
M2 segment	32 (10.2)	14 (9.9)	18 (5.8)	
Tandem-occlusion, no. (%)	22 (7.0)	12 (8.5)	10 (5.8)	0.370
Anesthesia, no. (%)				0.989
General	53 (16.9)	24 (16.9)	29 (17.0)	
Local	260 (83.1)	118 (83.1)	142 (83)	
hemisphere, no. (%)				0.503
Left	163 (52.1)	71 (50.0)	92 (53.8)	
Right	150 (47.9)	71 (50.0)	79 (46.2)	
intravenous thrombolysis, no. (%)	79 (25.2)	33 (23.2)	46 (26.9)	0.458
ASPECTS, no. (%)				0.558
3	69 (22.0)	28 (19.7)	41 (24.0)	
4	81 (25.9)	40 (28.2)	41 (24.0)	
5	163 (52.1)	74 (52.1)	89 (52.0)	
eTICI, no. (%)				0.001
2b	92 (29.4)	27 (19.0)	65 (38.0)	
2c	29 (9.3)	14 (9.9)	15 (8.8)	
3	192 (61.3)	101 (71.1)	91 (53.2)	

a Data on blood pressure on admission were missing (MR, 2/142 [1.4%] vs FR, 4/171 [2.3%]).

b Data on glucose were missing (MR, 1/142 [0.7%] vs FR, 5/171 [2.9%]).

c Data on last seen well to puncture time were missing (MR, 2/142 [1.4%] vs FR, 2/171 [1.2%]).

d Data on last seen well to recanalization time were missing (MR, 3/142 [2.1%] vs FR, 2/171 [1.2%]).

e Data on Puncture to recanalization time were missing (MR, 3/142 [2.1%] vs FR, 2/171 [1.2%]).

### 2.3 Statistical analysis

We performed statistical analyses using SPSS 29.0, employing Mann–Whitney U tests for continuous variables and χ^2^/Fisher’s exact tests for categorical variables. Significant univariate predictors (*p* < 0.05) and variables with established clinical relevance as key determinants of infarct progression and recanalization outcomes in ACLVO infarction were included in our multivariable logistic regression model (3, 14). The adjusted covariates comprised age, sex, history of atrial fibrillation, baseline NIHSS score, blood glucose (random admission glucose), ASITN/SIR score, and eTICI grade. The results of multivariable logistic regression were expressed as adjusted odds ratios (aOR) and 95% confidence intervals (CI). Missing data were minimal (0.6–2.9% per variable, see Table 1 footnotes) and handled via complete-case analysis, which is consistent with recommendations for negligible missingness (15). To evaluate the predictive performance of continuous variables in our multivariable logistic regression model, we generated individual ROC curves for each univariate predictor (age, glucose, NIHSS score) and multivariate combinations (age + baseline NIHSS, glucose + baseline NIHSS, glucose + age, age + glucose + NIHSS) using GraphPad Prism 10. The area under the curve (AUC) was used to measure both independent contributions and synergistic predictive effects. To explore potential synergistic effects, we generated distribution surface plots using SigmaPlot 15 to visualize predicted outcome probability variations. For subgroup analyses, we similarly incorporated variables meeting both statistical significance (*p* < 0.05) and clinical relevance into logistic regression models to explore the occurrence of FR.

## 3 Results

### 3.1 Patient characteristics

**Figure 1:**
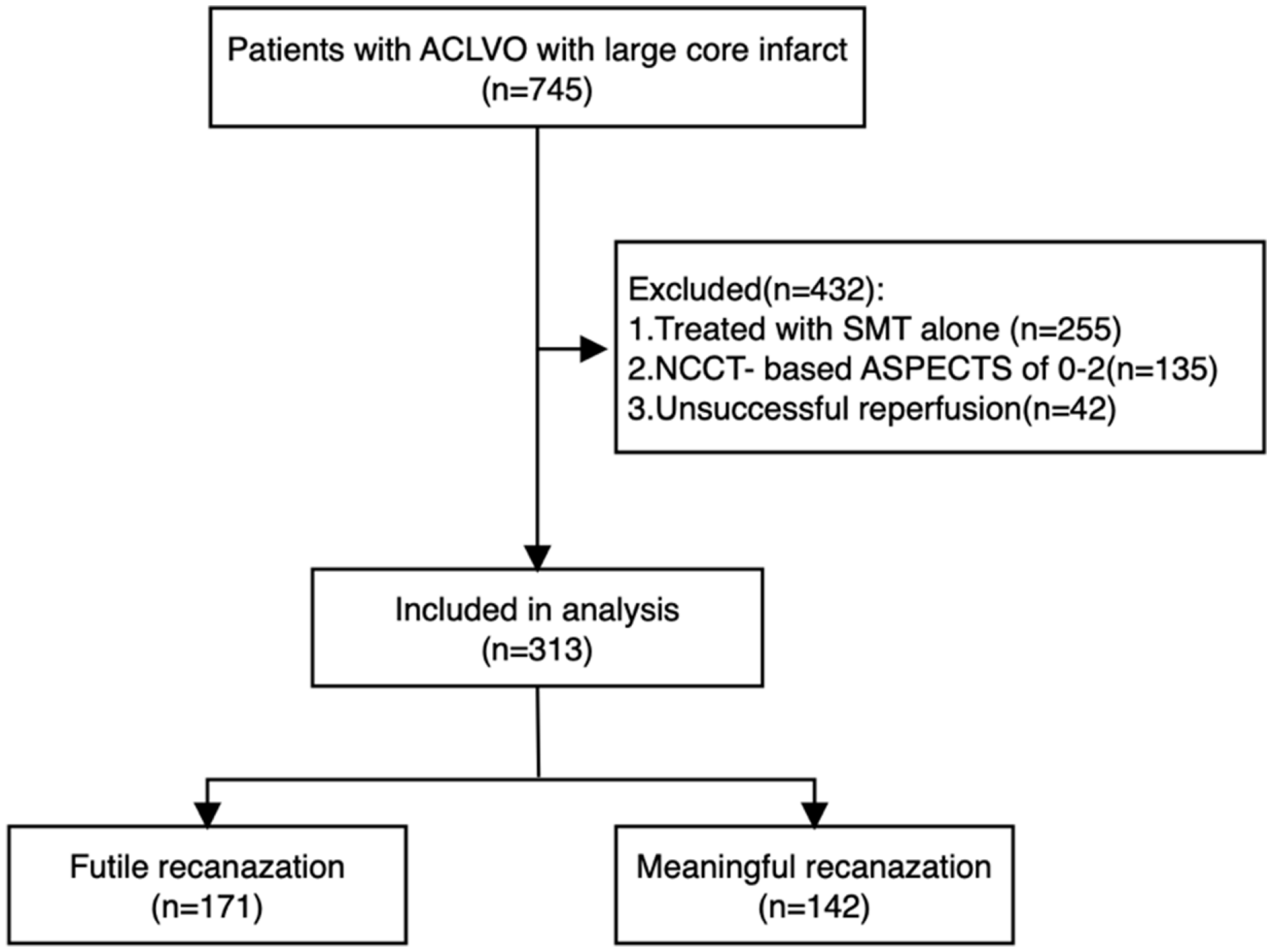
Flow diagram of our study. Flow diagram of our study. ACLVO, anterior circulation large vessel occlusion; SMT, standard medical treatment. NCCT-based ASPECTS, non-contrast computed tomography-Alberta Stroke Program Early CT Score.

The MAGIC registry collected data from 38 comprehensive stroke centers throughout China, a total of 745 eligible patients (November 2021 and February 2023). We excluded 255 patients who received only SMT, 135 patients with NCCT-based ASPECTS scores of 0–2, and 42 patients who did not achieve successful recanalization (eTICI ≥ 2b). Ultimately, 313 patients were included in the final analysis. Among these, 142 patients (45.4%) were categorized into the meaningful recanalization (MR) group, while 171 patients (54.6%) were classified into the FR group. The study flowchart is presented in Figure 1.

Compared with the MR group, patients in the FR group were significantly older, had higher blood glucose levels, and higher baseline NIHSS scores. Additionally, the FR group showed a greater proportion of male patients, a higher percentage of atrial fibrillation, and a worse distribution of ASITN/SIR grades and eTICI grades (Table 1).

### 3.2 Predictors of futile recanalization

A multivariate logistic regression model was developed to identify independent predictors of FR in patients who achieved successful recanalization after EVT. The analysis revealed that higher baseline NIHSS scores, older age, elevated blood glucose, poor collateral circulation, and incomplete recanalization were significantly associated with FR (Table 2).

**Table 2:** Multivariable analysis: predictors of futile recanalization.

Variables	Adjusted OR (95% CI)	*p* value
Age	1.058 (1.030–1.086)	<0.001
Glucose	1.209 (1.079–1.356)	0.001
ASITN/SIR grade		
0 ~ 1	1	0.001
2	0.406 (0.218–0.758)	0.005
3 ~ 4	0.275 (0.127–0.593)	<0.001
Baseline NIHSS score	1.132 (1.057–1.241)	<0.001
eTICI		
2b	1	<0.001
2c	0.305 (0.111–0.838)	0.021
3	0.296 (0.157–0.556)	<0.001

**Figure 2:**
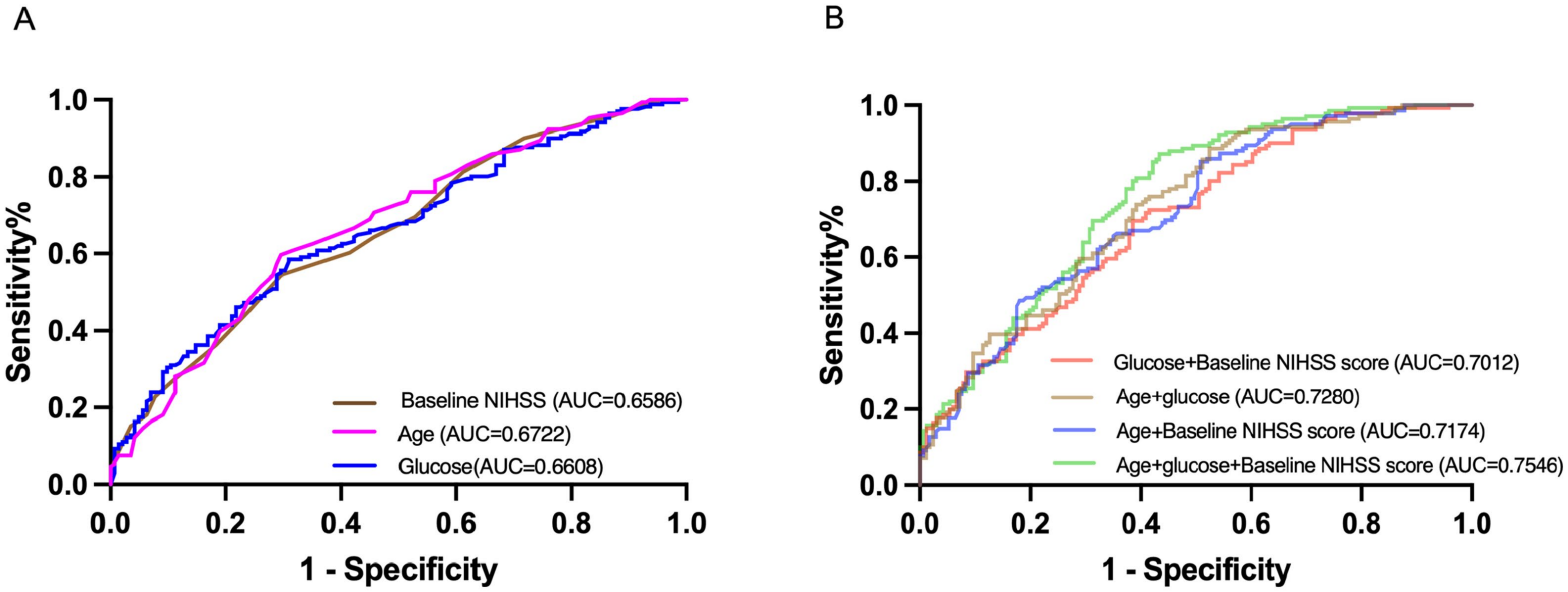
Receiver-operating characteristic curves. **(A)** Showed the single variables: baseline NIHSS, age, and glucose for futile recanalization. **(B)** Showed the multivariate variables: age + baseline NIHSS, age + glucose, glucose + baseline NIHSS, and age + glucose + baseline NIHSS for futile recanalization. AUC, area under the curve; NIHSS, National Institutes of Health Stroke Scale.

ROC analysis confirmed the predictive utility of these three independent predictors, with combined models showing improved discrimination (AUC = 0.7546, 95% CI, 0.7015–0.8078, *p* < 0.001) (Figure 2). To enhance clinical applicability, we derived optimal glucose cut-off values (7.5 mmol/L, which could effectively distinguish between MR and FR) using the maximum Youden’s index for subsequent subgroup analyses.

**Figure 3:**
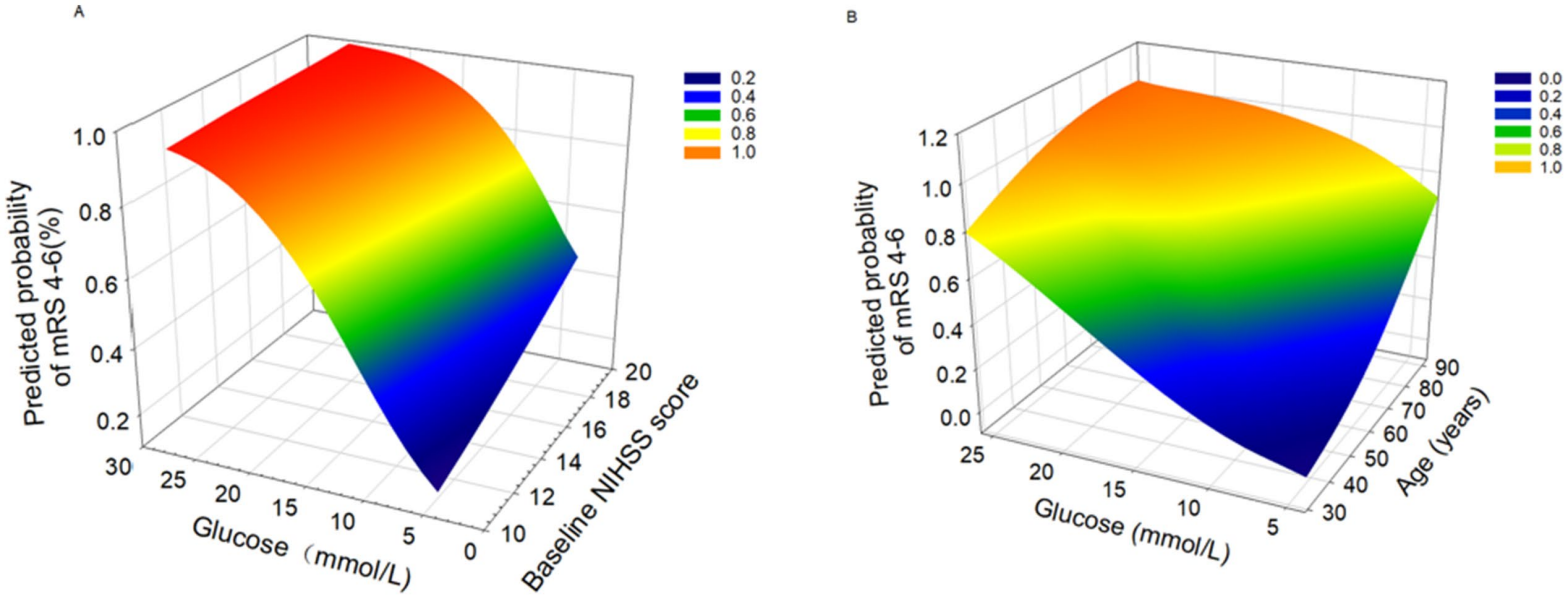
3D surface plots of predictors for futile rencanalization (defined as 90-day modified Rankin Scale [mRS] 4-6). **(A)** Association between glucose levels and baseline NIHSS score with predicted probability of mRS 4-6. **(B)** Association between glucose levels and age with predicted probability of mRS 4-6.

All variables in Figure 3 are continuous. The figure demonstrates the relationships between various predictors and the probability of FR. Figure 3A reveals a linear association between increasing blood glucose levels and higher FR probability when NIHSS scores are held constant. Notably, patients with higher NIHSS scores exhibit a steeper glucose-FR probability slope. Figure 3B demonstrates that concurrent elevations in both blood glucose and age are associated with increased probability of FR.

### 3.3 Subgroup analysis

Establishing population-specific treatment thresholds for blood glucose is critical. Hyperglycemia in hospitalized patients was defined as any glucose level ≥140 mg/dL (7.8 mmol/L) (16). We adopted the optimal predictive cutoff value (7.5 mmol/L, closely aligned to 7.8 mmol/L) identified in this analysis as the dichotomization threshold to explore the relationship and mechanisms between glucose levels and FR in our study.

After excluding 6 cases with missing data, the final cohort comprised 307 patients. We used the cutoff value (7.5 mmol/L) to stratify patients into two groups: low glucose (LG) group (glucose <7.5 mmol/L, *n* = 170) and high glucose (HG) group (glucose ≥7.5 mmol/L, *n* = 137). Univariate analysis results are presented in Supplementary Table 1.

In the multivariate logistic regression analysis, age was identified as an independent predictor in both groups. Additionally, in the LG group, higher baseline NIHSS scores were associated with FR. Interestingly, in the HG group, baseline NIHSS scores were not a predictor of FR. We also found that FR was significantly associated with ASITN/SIR grade in the HG group, whereas in the LG group, FR showed a correlation with the eTICI grade (Supplementary Table 2). We assessed collinearity between age, high glucose levels (glucose ≥ 7.5 mmoL/L), and collateral status (ASTIN/SIR grade). All VIF values were < 2 (age: 1.042, glucose: 1.000, collateral status: 1.042).

There were 94 (68.6%) patients with FR in the HG group, and 72 (42.4%) patients with FR in the LG group. We also found that among 260 (84.7%) non-diabetic patients and 47 (15.3%) diabetic patients in the cohort, the LG group, 7 (4.1%) patients had diabetes, with 5 (5.1%) cases in the MR group and 2 (2.8%) cases in the FR group. In contrast, the HG group contained 40 (29.2%) diabetic patients, comprising 13 (30.2%) in the MR group and 27 (28.7%) in the FR group. Notably, diabetes prevalence was 7-fold higher in the HG stratum (29.2% vs. 4.1%), yet diabetic status itself did not modify FR risk within either group (HG: 28.7% FR in diabetics vs. 30.2% MR; LG: 2.8% vs. 5.1%) (Supplementary Table 3). These findings suggest that acute hyperglycemia, rather than chronic diabetic status, drives FR.

## 4 Discussion

This study selected patients with ACLVO and large core infarction based solely on NCCT-based ASPECTS scores of 3–5. Our results demonstrated an FR incidence of 54.6%, closely mirroring the 53% reported by Huo et al. (3). However, their study enrolled patients based on Computed Tomography Perfusion (CTP). These findings suggest that functional outcomes are comparable between the two imaging modalities, and the findings align with prior research (17, 18). This does not, however, negate the potential benefits of advanced imaging techniques in improving outcomes. While advanced neuroimaging may improve patient selection for EVT (19, 20), strict reliance on these modalities risks excluding potentially eligible candidates, delaying treatment, and increasing the likelihood of unfavorable outcomes. Moreover, such imaging techniques remain inaccessible at many hospitals, particularly in resource-limited settings. In contrast, NCCT is widely available and routinely used in acute stroke workflows, offering a practical alternative for timely decision-making (21, 22).

Our study’s predictors: age, NIHSS score, eTICI grade, and ASITN/SIR grade are fundamental and strong in most models (23–25). Their predictive value is well-established: age reflects recovery capacity; NIHSS score quantifies baseline stroke severity. These factors are clinically easy to assess. Additionally, achieving higher eTICI grades directly improves outcomes. This is a key focus in neurointerventional practice. The mechanisms behind these predictors are clear and measurable. They provide reliable clinical guidance.

The relationship between acute hyperglycemia and FR remains controversial (26–28). Two large-scale RCTs have demonstrated that intensive perioperative glycemic control did not improve clinical outcomes (29, 30), which predominantly suggested no significant association between hyperglycemia and FR. However, our study supports a robust correlation between acute hyperglycemia and futile recanalization (FR), and these findings align with both the RESCUE-Japan LIMIT trial subgroup analysis and the conclusions of Tang et al.—despite some differences in specific thresholds and FR rates. Collectively, they point to a consistent trend regarding the relationship between glycemia and outcomes in such patients (16, 31). We argue that the negative results from glycemic control trials should not be interpreted as disproving a hyperglycemia-FR relationship, since treatment failure may reflect intervention timing rather than mechanism irrelevance. The rapid-onset pathological effects of acute hyperglycemia—including oxidative stress, inflammatory activation, and microthrombosis-likely cause irreversible damage before treatment initiation (32). Although current mechanistic understanding remains incomplete, these findings strongly support the need for further investigation into the underlying pathways.

Furthermore, the SELECT2 trial highlights that post-stroke care quality, rehabilitation strategies, and socioeconomic factors may significantly influence long-term patient outcomes. However, our current study is limited by the lack of longer-term longitudinal follow-up data (1-year or 3-year functional outcome). Future studies incorporating extended follow-up periods and comprehensive socioeconomic assessments are warranted to validate these observations.

### 4.1 Limitations

As with all multicenter prospective clinical studies, center-specific effects are inevitable. Due to the limited sample size in our study, we were unable to further validate the robustness of our findings using mixed-effects models, and the subanalysis’s results should also be interpreted with caution. Additionally, the lack of documentation regarding whether random admission blood glucose levels were measured under fasting conditions introduces a potential limitation to our subgroup analyses based on dichotomized cut-off values.

## 5 Conclusion

In conclusion, a significant proportion (54.6%) of ACLVO with large core infarct and NCCT-based ASPECTS scores of 3–5 experienced FR. The key predictors included higher NIHSS scores, older age, elevated blood glucose, poor collateral circulation, and incomplete recanalization. Further investigation into modifiable factors—particularly hyperglycemia’s role in reperfusion injury—may reveal therapeutic targets to reduce FR rates.

## Data Availability

The original contributions presented in the study are included in the article/Supplementary material, further inquiries can be directed to the corresponding authors.
