# Supplementary material for: Predictors of futile recanalization after endovascular therapy in anterior circulation stroke with large core infarction
Source: Front Neurol. 2025 Aug 20;16:1630438. doi: 10.3389/fneur.2025.1630438 (PMC12406493; doi:10.3389/fneur.2025.1630438)
Supplement: Supplementary file 1 [file Data_Sheet_1.docx]

**Supplementary Table1.****Baseline Characteristics in Patients with Meaningful and Futile** **Recanalization** **at Different Glucose Levels.**

|  | **Glucose <7.5mmol/L** | | | | **Glucose** **≥7.5mmol/L** | | | | | | |
| --- | --- | --- | --- | --- | --- | --- | --- | --- | --- | --- | --- |
|  | **Overall (n=170)** | **Meaningful recanalization** | **Futile recanalization** | **P** | **Overall (n=137)** | | **Meaningful recanalization** | | **Futile recanalization** | **P** | |
| Age, median (IQR), y | 65.4(56-76.3) | 62.4(53-75) | 69.4(60-78.8) | 0.002 | | 70(62-78) | | 64(57-69) | 72.7(66.8-80) | <0.001 |  |
| Sex, no.(%) |  |  |  | 0.017 | |  | |  |  | 0.605 |  |
| Men | 116(68.2) | 74(75.5) | 42(58.3) |  | | 72(52.6) | | 19(44.2) | 46(48.9) |  |  |
| Women | 54(31.8) | 24(24.5) | 30(46.2) |  | | 65(47.4) | | 24(55.8) | 48(51.1) |  |  |
| Baseline NIHSS score, median (IQR) | 16(13-19) | 15(12-18) | 17(14-20) | 0.001 | | 17(13-20) | | 16(13-19) | 19(14-22) | 0.004 |  |
| Medical history, no. (%) |  |  |  |  | |  | |  |  |  |  |
| Hypertension | 97(57.1） | 54(55.1) | 43(59.7) | 0.548 | | 91(66.4) | | 27(62.8) | 64(68.1) | 0.543 |  |
| Hyperlipidemia | 41(24.1) | 23(23.5) | 18(25) | 0.818 | | 28(20.4) | | 10(23.3) | 18(19.1) | 0.580 |  |
| Diabetes | 7(4.1) | 5(5.1) | 2(2.8) | 0.451 | | 40(29.2) | | 13(30.2) | 27(28.7) | 0.857 |  |
| Smoking | 73(42.9) | 43(43.9) | 30(41.7) | 0.774 | | 31(22.6) | | 12(27.9) | 19(61.3) | 0.318 |  |
| Atrial fibrillation | 71(41.8) | 36(36.7) | 35(48.6) | 0.121 | | 67(48.9) | | 17(39.5) | 50(53.2) | 0.138 |  |
| Blood pressure on admission,  median (IQR), mmHg ^a^ | |  |  |  | |  | |  |  |  |  |
| Systolic | 143(125-158) | 143(125-158) | 144(133-160) | 0.774 | | 147(129-166) | | 142(127-161) | 150(130-169) | 0.104 |  |
| Diastolic | 85(75-94) | 85(75-95) | 83(73-94) | 0.331 | | 85(77-91) | | 85(76-94) | 86(77-91) | 0.898 |  |
| Stroke causative  mechanism, no. (%) |  |  |  | 0.738 | |  | |  |  | 0.462 |  |
| Large artery. Atherosclerosis | 47(27.6) | 29(29.6) | 18(25) |  | | 48(35) | | 19(44.2) | 29(30.9) |  |  |
| Cardioembolism | 99(58.2) | 54(55.1) | 45(62.5) |  | | 75(54.7) | | 21(48.8) | 54(57.4) |  |  |
| Other | 7(4.1) | 5(5.1) | 2(2.8) |  | | 4(2.9) | | 1(2.3) | 3(3.2) |  |  |
| Unknow | 17(10) | 10(10.2) | 7(9.7) |  | | 10(7.3) | | 2(4.7) | 8(8.5) |  |  |
| ASITN/SIR grade, no. (%) |  |  |  | 0.160 | |  | |  |  | <0.001 |  |
| 0-1 | 65(38.2) | 32(32.7) | 33(45.8) |  | | 66(48.2) | | 9(20.9) | 57(60.6) |  |  |
| 2 | 76(44.7) | 46(46.9) | 30(41.7) |  | | 43(31.4) | | 19(44.2) | 24(25.5) |  |  |
| 3-4 | 29(17.1) | 20(20.4) | 9(12.5) |  | | 28(20.4) | | 15(34.9) | 13(13.8) |  |  |
| Last seen well to imaging time, median (IQR), min | 267(158-471) | 255(150-386) | 296(168.8-522.5) | 0.136 | | 323(163-437) | | 304(190.5-392) | 325.5(143-446.3) | 0.985 |  |
| Last seen well to puncture time, median (IQR), min ^b^ | 341(240-600) | 327(240-532) | 370(251-612.5) | 0.325 | | 390(237-523) | | 383(287.3-514) | 390(227.5-530.5) | 0.757 |  |
| Last seen well to recanalization time, median (IQR), min ^c^ | 427(316-693) | 420(325-674) | 431.5(298-732.5) | 0.696 | | 455(321-630) | | 461(351-570.5) | 453(307.5-644.5) | 0.84 |  |
| Puncture to recanalization time, median (IQR),min ^d^ | 88.3(50-112.3) | 80.3(50-103) | 99.6(50-123.8) | 0.066 | | 90.1(55-119) | | 82.8(45-112) | 93(59-120) | 0.117 |  |
| First choice of endovascular treatment |  |  |  | 0.457 | |  | |  |  | 0.470 |  |
| Stent retriever thrombectomy | 37(21.8) | 21(21.4) | 16(22.2) |  | | 21(15.3) | | 7(16.3) | 14(14.9) |  |  |
| Aspiration | 94(55.3) | 53(54.1) | 41(56.9) |  | | 85(62) | | 30(69.8) | 55(58.5) |  |  |
| Balloon angioplasty and/or stenting | 9(5.3) | 5(5.1) | 4(5.6) |  | | 10(7.3) | | 4(9.3) | 6(6.4) |  |  |
| Intra-arterial medication and/or mechanical fragmentation | 3(1.8) | 1(1) | 2(2.8) |  | | 2(1.4) | | 0(0) | 2(2.2) |  |  |
| Swim | 26(15.3) | 18(18.4) | 8(11.1) |  | | 18(13.1) | | 2(4.7) | 16(17) |  |  |
| Spontaneous reperfusion | 1(0.5) | 0(0) | 1(1.4) |  | | 1(0.7) | | 0(0) | 1(1.1) |  |  |
| Pass stent no. (%) |  |  |  | 0.086 | |  | |  |  | 0.584 |  |
| <3 | 164(90.3) | 96(99) | 68(94.4) |  | | 130(96.3) | | 41(97.6) | 89(95.7) |  |  |
| ≥3 | 5(9.7) | 1(1) | 4(5.6) |  | | 5(3.7) | | 1(2.4) | 4(4.3) |  |  |
| Pass aspiration, no. (%) |  |  |  | 0.081 | |  | |  |  | 0.882 |  |
| <3 | 165(97.6) | 93(95.9) | 72(100) |  | | 126(93.3) | | 39(92.9) | 87(93.5) |  |  |
| ≥3 | 4(2.4) | 4(4.1) | 0(0) |  | | 9(6.7) | | 3(7.1) | 6(6.5) |  |  |
| Occlusion-site, no. (%) |  |  |  | 0.827 | |  | |  |  | 0.122 |  |
| Internal carotid artery | 46(27.1) | 25(25.5) | 21(29.2) |  | | 59(43.1) | | 14(32.6) | 45(47.9) |  |  |
| M1 segment | 108(63.5) | 63(64.3) | 45(62.5) |  | | 62(45.3) | | 25(58.1) | 37(39.4) |  |  |
| M2 segment | 16(9.4) | 10(10.2) | 6(8.3) |  | | 16(11.7) | | 4(9.3) | 12(12.8) |  |  |
| Tandem-occlusion, no. (%) | 11(6.5) | 6(6.1) | 5(6.9) | 0.830 | | 11(8) | | 6(14) | 5(5.3) | 0.840 |  |
| Anesthesia, no. (%) |  |  |  | 0.391 | |  | |  |  | 0.182 |  |
| General | 26(15.3) | 13(13.3) | 13(13.3) |  | | 26(19) | | 11(25.6) | 15(16) |  |  |
| Local | 144(84.7) | 85(86.7) | 59(86.7) |  | | 111(81) | | 32(74.4) | 79(84) |  |  |
| Hemisphere, no. (%) |  |  |  | 0.886 | |  | |  |  | 0.218 |  |
| Left | 91(53.5) | 52(53.1) | 39(54.2) |  | | 68(49.6) | | 18(41.9) | 50(53.2) |  |  |
| Right | 79(46.5) | 46(46.9) | 33(45.8) |  | | 69(50.4) | | 25(58.1) | 44(46.8) |  |  |
| Intravenous thrombolysis, no. (%) | 38(22.4) | 19(19.4) | 19(26.4) | 0.279 | | 41(29.9) | | 14(32.6) | 27(28.7) | 0.649 |  |
| ASPECTS, no. (%) |  |  |  | 0.679 | |  | |  |  | 0.070 |  |
| 3 | 37(21.8) | 22(22.4) | 15(20.8) |  | | 30(21.9) | | 6(14) | 24(25.5) |  |  |
| 4 | 46(27.1) | 24(24.5) | 22(30.6) |  | | 35(25.5) | | 16(37.2) | 19(20.2) |  |  |
| 5 | 87(51.2) | 52(53.1) | 35(48.6) |  | | 72(52.6) | | 21(48.8) | 51(54.3) |  |  |
| eTICI, no. (%) |  |  |  | 0.002 | |  | |  |  | 0.162 |  |
| 2b | 49(28.8) | 18(18.4) | 31(43.1) |  | | 43(31.4) | | 9(20.9) | 34(36.2) |  |  |
| 2c | 16(9.4) | 11(11.2) | 5(6.9) |  | | 11(8) | | 3(7) | 8(8.5) |  |  |
| 3 | 105(61.8) | 69(70.4) | 36(50) |  | | 83(60.6) | | 31(72.1) | 52(55.3) |  |  |

^a^ Data on blood pressure on admission were missing (Glucose <7.5mmol/L, MR, 2/98 [2%] vs FR, 1/72 [1.4%]; Glucose ≥7.5mmol/L, MR, 0/43 [0%] vs FR, 2/94 [2.1%]).

^b^ Data on last seen well to puncture time were missing (Glucose <7.5mmol/L, MR, 1/98 [1.4%] vs FR, 1/72 [1.4%]; Glucose ≥7.5mmol/L, MR, 1/43 [2.3%] vs FR,1/94 [1.1%]).

^c^ Data on last seen well to recanalization time were missing (Glucose <7.5mmol/L, MR, 2/98 [2%] vs FR, 1/72 [1.4%]; Glucose ≥7.5mmol/L, MR, 1/43 [2.3%] vs FR,1/94 [1.1%]).

^d^ Data on Puncture to recanalization time were missing (Glucose <7.5mmol/L, MR, 2/98 [2%] vs FR, 1/72 [1.4%]; Glucose ≥7.5mmol/L, MR, 1/43 [2.3%] vs FR,1/94 [1.1%]).

**Supplementary Table 2:** **Subgroup Multivariable Analysis: Predictors of Futile Recanalization.**

|  | **Glucose <7.5mmol/l** | | **Glucose** **≥7.5mmol/l** | |
| --- | --- | --- | --- | --- |
|  | **Adjusted OR (95% CI)** | **P Value** | **Adjusted OR (95% CI)** | **P Value** |
| Age | 1.038(1.009-1.069) | 0.010 | 1.072(1.028-1.118) | 0.001 |
| Baseline NIHSS score | 1.171(1.069-1.284) | <0.001 | 1.093(0.990-1.207) | 0.079 |
| eTICI |  |  |  |  |
| 2b | 1 | 0.002 | NA | NA |
| 2c | 0.218(0.059-0.814) | 0.023 | NA | NA |
| 3 | 0.253(0.116-0.550) | <0.001 | NA | NA |
| ASITN/SIR grade |  |  |  |  |
| 0-1 | NA | NA | 1 | 0.002 |
| 2 | NA | NA | 0.212（0.079-0.572） | 0.002 |
| 3-4 | NA | NA | 0.170（0.055-0.518） | 0.002 |

NA, variables with *P* > .05 in univariate analysis were excluded from regression.

**Supplementary Table 3: Futile Recanalization between Diabetic and Non-diabetic Patients at Different Glucose Levels.**

| **Variable** | **Glucose <7.5mmol/l**  **(n=170)** | **Glucose≥7.5mmol/l**  **(n=137)** | **Total (N=307)** | **P Value** |
| --- | --- | --- | --- | --- |
| \| FR, n. (%) \| \| --- \| | 72 (42.4%) | 94 (68.6%) | 166 (54.1%) | <0.001 |
| Diabetes, n. (%) |  |  |  | <0.001 |
| No | 163 (95.9%) | 97 (70.8%) | 260 (84.7%) |  |
| Yes | 7 (4.1%) | 40 (29.2%) | 47 (15.3%) |  |
| FR with diabetes, n/N (%) |  |  |  | 0.451^a^ vs 0.843^b^ |
| No | 70/163 (42.9%) | 67/97 (69.1%) | 137/260 (52.7%) |  |
| Yes | 2/7 (28.6%) | 27/40 (67.5%) | 29/47 (61.7%) |  |

FR, futile recanalization; ^a^ Group with Glucose<7.5mmol/l; ^b^ Group with Glucose≥7.5mmol/l.
